# Juxtaposition between host population structures: implications for disease transmission in a sympatric cervid community

**DOI:** 10.1111/eva.12065

**Published:** 2013-10-09

**Authors:** Eric Vander Wal, Iain Edye, Paul C Paquet, David W Coltman, Erin Bayne, Ryan K Brook, José A Andrés

**Affiliations:** 1 Department of Biology, University of SaskatchewanSaskatoon, SK, Canada; 2 Department of Biological Sciences, University of AlbertaEdmonton, Alberta, Canada; 3 Raincoast Conservation FoundationDenny Island, BC, Canada; 4 Department of Animal and Poultry Science and Indigenous Land Management Institute, College of Agriculture and Bioresources, University of SaskatchewanSaskatoon, SK, Canada

**Keywords:** Cervidae, community, Discriminant Analysis of Principal Components, disease transmission, *Mycobacterium bovis*, population genetics, spatial Principal Component Analysis, sympatric populations

## Abstract

Sympatric populations of phylogenetically related species are often vulnerable to similar communicable diseases. Although some host populations may exhibit spatial structure, other hosts within the community may have unstructured populations. Thus, individuals from unstructured host populations may act as interspecific vectors among discrete subpopulations of sympatric alternate hosts. We used a cervid-bovine tuberculosis (*Mycobacterium bovis*) system to investigate the landscape-scale potential for bovine tuberculosis transmission within a nonmigratory white-tailed deer (*Odocoileus virginianus*) and elk (*Cervus canadensis*) community. Using landscape population genetics, we tested for genetic and spatial structure in white-tailed deer. We then compared these findings with the sympatric elk population that is structured and which has structure that correlates spatially and genetically to physiognomic landscape features. Despite genetic structure that indicates the white-tailed deer population forms three sympatric clusters, the absence of spatial structure suggested that intraspecific pathogen transmission is not likely to be limited by physiognomic landscape features. The potential for intraspecific transmission among subpopulations of elk is low due to spatial population structure. Given that white-tailed deer are abundant, widely distributed, and exhibit a distinct lack of spatial population structure, white-tailed deer likely pose a greater threat as bovine tuberculosis vectors among elk subpopulations than elk.

## Introduction

One of the key goals of disease ecology is to understand pathogen transmission and disease spread over space and time (Tompkins et al. 2011). To date, the majority of research to describe and predict the spread of disease in natural populations is based on the transmission heterogeneity within a single host population. However, in many cases, pathogens are not species-specific and pathogen transmission is a multi-host community-level phenomenon (Rigaud et al. 2010). Ignoring the interactions among different members of the host community greatly limits our ability to estimate different epidemiological parameters (McCoy et al. 2003; Johnson and Thieltges 2010; Searle et al. 2011). Identifying the relative contributions of different host species, their interactions, and environmental characteristics in overall transmission heterogeneity is clearly needed (Paull et al. 2012).

Studying multi-host endemic disease systems is often complicated because they commonly involve highly mobile and widely distributed wildlife species with low prevalence of infection [e.g., chronic wasting disease, CWD and bovine tuberculosis, *Mycobacterium bovis,* bTB (Conner et al. 2008)]. As a result, in these systems, empirical data from which to derive or apply traditional epidemiological models are lacking. Professionals responsible for managing and preventing the spread of disease need to employ alternative methods to infer potential infection pathways and disease dynamics. This is particularly important in cases where the social and economic ramifications of the disease are unrelated to its prevalence, but rather just its presence or absence [see, e.g., the impact of bTB on beef exports in Manitoba (CAN), Nishi et al. (2006)]. In these cases, population genetics is a useful method to estimate the potential for disease spread by revealing the permeability (ease with which animals can penetrate and pass through) of specific landscapes (Biek and Real 2010, Remais et al. 2011). This genetic approach is appropriate for free-ranging and unbounded wildlife populations and has been recently applied to understand and predict landscape-scale transmission of several pathogens in natural populations including CWD (Blanchong et al. 2008; Cullingham et al. 2011a,b), bTB (Blanchong et al. 2007; Vander Wal et al. 2012), and raccoon rabies (Cullingham et al. 2009; Côté et al. 2012) by examining only one of several potential hosts in the system. However, this approach has not explicitly been considered to study multiple hosts simultaneously. Even if phylogenetically similar, hosts may exhibit very different behaviors relating to intraspecific sociality, movement rates, philopatry, habitat, and resource requirements. Therefore, landscape permeability for one host may differ from heterospecifics, resulting in one host acting as a vector among allopatric demes of the alternate host species. In this study, we aimed to expand upon this single host population approach (see Fenton et al. 2002) and apply new genetic methods to compare the potential pathogen transmission at a broad landscape scale between two largely sympatric bTB hosts, white-tailed deer (*Odocoileus virginianus*) and elk (*Cervus canadensis*).

Bovine tuberculosis is a generalist pathogen affecting a wide range of species globally (Daszak et al. 2000), including two members of a cervid community in southwestern Manitoba (Canada) (Nishi et al. 2006; Brook 2009). In this region, elk and white-tailed deer are abundant free-ranging hosts for bTB [although susceptible (Hawden 1942), bTB has not been detected in the third member of the community, moose (*Alces alces*), despite widespread testing (Parks Canada unpublished data)]. Limited connectivity among elk subpopulations in the region indicates a low potential for long-distance disease spread through the movement and dispersal of infected elk (Vander Wal et al. 2012). However, the potential for long-distance (i.e., >30 km to the nearest large protected area) disease spread within and between elk and white-tailed deer populations may persist through the dispersals of white-tailed deer from the infection focus. Several genetic studies on single host-pathogen systems suggest that this might be the case (Blanchong et al. 2008; Cullingham et al. 2011a; Lang and Blanchong 2012). Landscape genetics of white-tailed deer has been well studied (Mathews and Porter 1993; Scribner et al. 1997; Blanchong et al. 2006; Comer et al. 2011; Miller et al. 2011; Robinson et al. 2012), particularly with reference to two critical infectious agents, bTB (Blanchong et al. 2007) and CWD (Blanchong et al. 2008; Grear et al. 2010; Cullingham et al. 2011a). In contrast to our findings for elk (Vander Wal et al. 2012), these studies showed that high landscape permeability for white-tailed deer with very little differentiation among populations suggesting relatively frequent long-distance movements and a high potential for regional disease dispersal (Cullingham et al. 2011a).

Herein, we combine a multi-host population genetics approach with bTB distribution data to test whether white-tailed deer, considered to be a secondary host in this region, are more likely to be involved in long-distance transmission of bTB outside of the endemic area. To do so, we test first for genetic and spatial structure in the white-tailed deer population. We then augment results from Vander Wal et al. (2012) with a new analysis of additional spatial data to reaffirm that the elk population is spatially structured. If the white-tailed deer population is unstructured, as we predict, our system will provide the necessarily juxtaposition between population structures to test whether population structure affects the dispersion of bTB-infected white-tailed deer and elk. We predict that an unstructured white-tailed deer population will result in higher dispersion of bTB-infected individuals than a structured elk population.

## Materials and methods

### Study area

**Figure 1:**
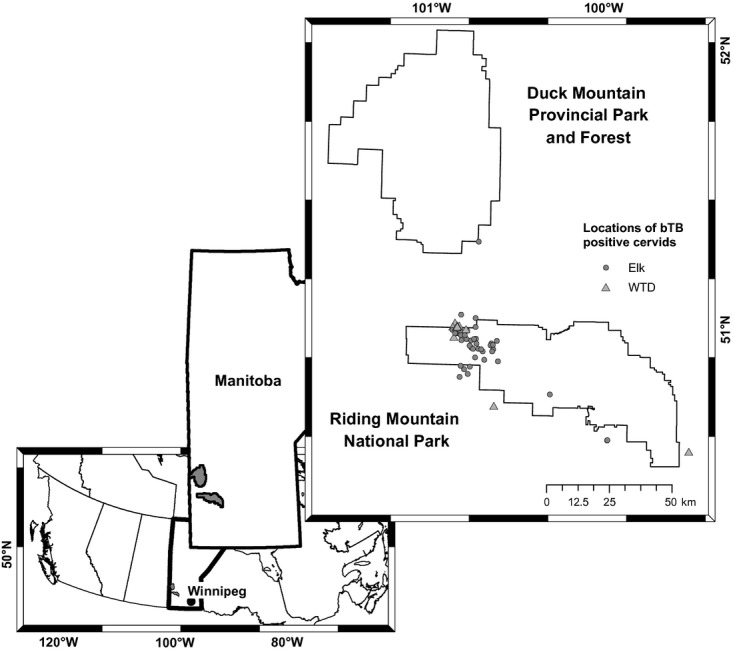
Riding Mountain National Park (RMNP) and Duck Mountain Provincial Park and Forest (DMPP&F), Manitoba, CAN with spatial locations of bovine tuberculosis (bTB)-positive white-tailed deer (WTD; *n* = 11) and elk (*n* = 41) and from 1991 to 2010 illustrating apparent disease clustering in the Riding Mountain Region.

In southwestern Manitoba, Canada, the Boreal Plains ecoregion (Bailey 1968) transitions into the Prairie ecoregion (Olson et al. 2001). This area includes the Riding Mountain region, which is comprised of Riding Mountain National Park (RMNP; 3000 km^2^) and the Duck Mountain Provincial Park and Forest (DMPP&F 3800 km^2^) (Fig. 1). The region encompasses the Manitoba Lowlands and the Manitoba Escarpment, resulting in a 475 m elevation change. The altitudinal gradient results in variation in vegetation (Caners and Kenkel 2003) and in local climate. An agriculture-dominated matrix surrounds both reserves. The matrix acts as a barrier to elk movement (Vander Wal et al. 2012) and elk are predominantly within the protected areas (Brook 2008); however, white-tailed deer occur in both reserves and are also abundant in the surrounding agricultural matrix (Brook et al. 2012). Over the last five decades, agricultural expansion has eroded a once extensive forest and native grassland corridor between RMNP and DMPP&F (Walker 2001).

The occurrence of bTB is clumped spatially (Fig. 1) and corresponds largely to the distribution of elk subpopulations; however, it is not restricted to elk. Extensive testing of a wide range of other potential bTB hosts in the region has not identified any other infected wildlife species (Parks Canada unpublished data). The apparent prevalence of bTB differs markedly between elk and white-tailed deer. Apparent prevalence of bTB in white-tailed deer is estimated at <1% (Nishi et al. 2006; Wobeser 2009). Conversely, of the 41 elk detected with bTB (1991–2010), 37 have been reported in the west subpopulation of RMNP, that is, 2.7% apparent prevalence (Shury and Bergeson 2011). Fourteen cattle (*Bos taurus*) herds around RMNP have tested positive for bTB between 1991 and 2010 (Shury and Bergeson 2011), which has resulted in significant socioeconomic repercussions and conflicts (Brook and McLachlan 2006; Nishi et al. 2006; Brook et al. 2012).

### General sampling considerations

For this study, we used two different sampling techniques to collect white-tailed deer and elk genetic samples. Elk samples were collected through the winter using a net gun fired from a helicopter (see Vander Wal et al. 2012 for sampling details). Most of the white-tailed deer samples were collected from autumn hunter kills. Although homogeneity in sampling methods is always desirable, this was logistically unfeasible. In such cases, combining genetic data from multiple sources is common, including hunt-harvested samples (e.g., Blanchong et al. 2008; Grear et al. 2010; Cullingham et al. 2011a,b; Rogers et al. 2011). Theoretically, structure artifacts may arise if the hunted individuals have disrupted home ranges and/or if hunter chased the animals for some distance. However, at a broad regional scale (>40 000 km^2^ in this study), the effects of hunting in the estimates of population subdivision are likely to be negligible. Also critical is obtaining sufficiently large and widespread distribution of the samples used. In our study, both strategies yielded widespread and large samples sizes.

### White-tailed deer sampling

We sampled tissues from 494 individuals between mid-October and early December (2004–2006) following the animal care protocol 472602 of the University of Alberta. Although most of the samples (≍94%, *n =* 464) came from hunt-harvested deer, a few of them (≍6%, *n =* 30) were obtained by Parks Canada using a helicopter-deployed net gun (see Cattet et al. 2004; Shury and Bergeson 2011 for details). Total genomic DNA was extracted using the standard Qiagen DNeasy protocol. Each sample was genotyped at 24 microsatellite loci using a series of PCR with fluorescence-labeled primers (Anderson et al. 2002; Supplementary Table S1). Resulting products were run in an ABI-3730 genetic analyzer using 600 LIZ as internal standard. Alleles were then sized using GeneMapper 4.0 software (Applied Biosystems Inc., Foster City, CA, USA).

### White-tailed deer population structure analyses

Statistical descriptors such as, allele diversity, expected (*H*_E_) and observed (*H*_O_) heterozygosity estimates, and tests of Hardy–Weinberg equilibrium (HWE) were obtained using the ADEGENET package (Jombart 2008). Natural populations often possess complex genetic structures that are not always well described by explicit, hierarchical genetic models. Thus, we examined the genetic differentiation of white-tailed deer using nonmodel (i.e., principal component analysis; PCA)-based approaches comparable to those previously used in the juxtaposed population of elk (Vander Wal et al. 2012). Unlike Bayesian clustering (Pritchard et al. 2000; Guillot et al. 2005; Corander et al. 2008), these methods do not rely on explicit population genetics models, and they are preferable when many loci are available and the structure is subtle (Jombart et al. 2008, 2010; Reeves and Richards 2009). Specifically, we used Discriminant Analysis of Principal Components [DAPC (Jombart et al. 2010)] and Spatial Principal Component Analysis [sPCA (Jombart et al. 2008)] as implemented in ADEGENET (R.2.11.1; R Development Core Team 2011). These multivariate methods are designed to reveal groups of genetically related individuals directly from genetic polymorphism data, rather than on notions of existing structure that are reliant on the assumptions of HWE.

DAPC transforms data using a principal component (PC) analysis before summarizing genetic variance between and within groups [i.e., a discriminant analysis (DA) (Jombart et al. 2010)]. The optimal number of clusters (i.e., *K*, demes) is inferred using sequential *K*-means and model selection (Jombart et al. 2010). Because at broad regional scale (>1000 km^2^) spatial population structure, white-tailed deer are typically panmictic (*K* = 1; Mathews and Porter 1993; Blanchong et al. 2008; Grear et al. 2010; Cullingham et al. 2011a), we varied *K* from 1 to 20 extending our analyses far beyond the number of populations that might be expected (Robinson et al.^2012^). We identified the optimal *K* as the one showing the lowest Bayesian Information Criterion (BIC). Then we used DAPC to assign individuals into populations, retaining the number of principal components using 85% of the cumulative deviance.

Spatial PCA summarizes spatial patterns of genetic structure by defining eigenvalues that optimize the product of the genetic variance and Moran's *I* (Moran 1948, 1950). Patterns are divided into positive (i.e., global) and negative (i.e., local), such that global patterns are used to identify clines in allele frequencies and genetically distinguishable groups. Conversely, local patterns detect differences between nearby individuals (Jombart et al. 2008). For further descriptions of the sPCA analyses see Vander Wal et al. (2012). Variation among and within the predicted clusters was estimated using Analysis of Molecular Variance (amova), which was implemented in Arlequin (Excoffier and Lischer 2010).

### Elk population structure analyses

The methods used to analyze white-tailed deer (e.g., sPCA) were also used in a recent population genetics study of the sympatric elk population, which comprises at least three spatially and genetically distinct clusters (Vander Wal et al. 2012). This suggested a low potential for long-distance disease spread through the movement and dispersal of infected elk. Hence, to further assess connectivity, we contrasted our previous population genetics findings with a large radio-telemetry dataset (*n =* 11 194 locations *n* = 379 elk collected from 2002–2009). Free-ranging elk were captured with a net gun from a helicopter (Cattet et al. 2004) following the animal care protocols #F01-037 (University of Manitoba) and #20060067 (University of Saskatchewan). Elk were relocated 1–16 times per fortnight using a fixed-wing aircraft (Cessna 172; Wichita, KS, USA) and ground telemetry and their position entered into a geographic positioning system (see Vander Wal et al. 2011 for details). Minimum convex polygons (95%) were chosen to delineate the borders of the subpopulations and assigned using the Home Range Tools (Rodgers et al. 2007) extension in ArcGIS (Redlands, CA, USA).

### Bovine tuberculosis testing in white-tailed deer and elk

A total of 6909 white-tailed deer and 3620 elk were tested for bTB infection status using a two-stage process (Shury and Bergeson 2011). First, lymphocyte stimulation test, fluorescence polarization assay, and chromatographic immunoassay were used in parallel to ascertain whether animals were suspected of being infected with bTB. Subsequently, suspected individuals were recaptured and killed with a captive bolt gun according to the guidelines of the Canadian Council for Animal Care and had tissues harvested and cultured to confirm or refute initial assays. For further information on disease testing protocols see Rousseau and Bergeson (2005) and Shury and Bergeson (2011), and for assay descriptions see Rohonczy et al. (1996) and Surujballi et al. (2009).

### Dispersion of bovine tuberculosis-positive white-tailed deer and elk

Broad-scale population structure is likely to affect the probability of landscape-scale disease transmission. In contrast to the relatively structured population of elk, we expected the white-tailed deer population to be panmictic (or quasi-panmictic). Thus, we predicted infected individuals would be more geographically dispersed than infected elk. To test this hypothesis, we used the spatial location of bTB-positive white-tailed deer (*n* = 11) and elk (*n* = 41; Fig. 1) collected between 1991 and 2011 to test whether bTB-positive white-tailed deer were more dispersed that bTB-positive elk. We used two approaches to test for differences in dispersion of diseased animals. First, we compared the mean nearest-neighbor distances between bTB-positive individuals with a Mann–Whitney *U*-test because sample sizes were unequal and distributions non-normal. Second, to control for differences in the distribution of sampled elk and white-tailed deer, we randomly sampled the locations of 11 bTB-positive elk (without replacement) and subsequently calculated their mean nearest-neighbor distances. This was repeated 1000 times, and the means were pooled to create a bootstrap distribution against which we could test the average mean nearest-neighbor distance of bTB-positive white-tailed deer. We programed analyses in R v 2.11 (R Development Core Team 2011).

## Results

### White-tailed deer: population and spatial structure

Our full dataset consisted of 24 microsatellite loci scored for 494 white-tailed deer drawn from RMNP and the Duck Mountain Provincial Park and Forest. We ran analyses on the full dataset and a subset of microsatellites (*n* = 17 microsatellites). The subset excluded those with proportions of missing data > 0.06 (*n* = 3 microsatellites). When we applied a Bonferroni correction for multiple comparisons, no loci exhibited significant deviation from HWE (at *P* < 0.0001, Table S1 in the Supplementary Material). However, using a more conservative approach, we also ran our analysis excluding loci that were not at HWE at *P* < 0.05 (an additional *n* = 4 microsatellites; Table S1 in the Supplementary Material). Results did not change among subsets and all failed to detect spatial structure (below).

**Figure 2:**
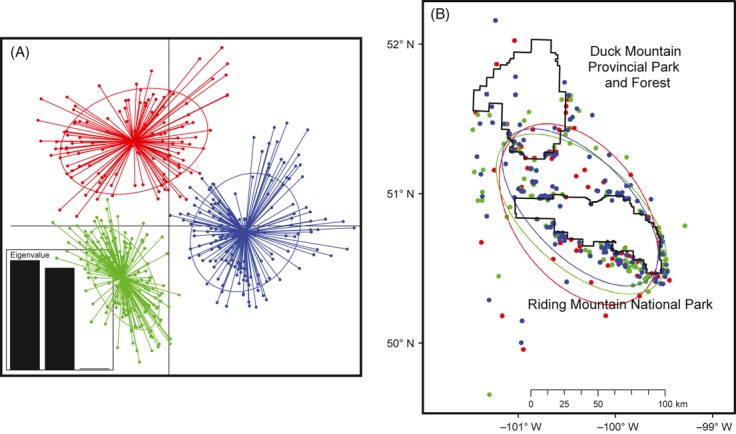
Discriminant Analysis of Principal Components for the 494 genotyped white-tailed deer from Riding Mountain National Park (2004–2006) revealed three genetically distinct (A) clusters on the landscape. However, as indicated by overlapping ellipses (B) clusters (red, green, blue) are sympatric.

The average numbers of alleles per locus was 12 (range from 2 to 23). The mean *H*_*O*_ across loci (0.627) was lower than the mean *H*_*E*_ (0.730). Almost all loci (22/24) showed an excess of homozygotes and significant deviations from HWE (*P* < 0.05) occurred in 5/24 loci, suggesting the existence of inbreeding and/or population subdivision (i.e., Wahlund effect). Therefore, we used DAPC to investigate the partition of genetic variation in this dataset. Our analyses showed that the BIC reached its minimum value at *K* = 3 and consequently displayed the smallest increase from *K* = 3 to *K* = 4. This strongly suggested that population subdivision into three clusters should be considered. We retained 75 principal components of PCA in the preliminary data transformation step, which altogether contained more that 85% of the total genetic variation. The first two principal components of DAPC explained 10% of this variation and were sufficient to capture the genetic structure of the white-tailed deer population (Fig. 2A). The first principal component differentiated cluster 3 (blue) from clusters 1 and 2 (green and red), whereas the second principal component displayed the genetic difference between cluster 2 (red) and the other two. The mean cluster membership probabilities based on the retained discriminant functions were >0.96, and only ≍7% of the individuals (35/494) showed some traces of admixture (i.e., no more than 80% membership in a single cluster). Our results, however, clearly showed no correspondence between genetic and spatial structures. DAPC results suggest no spatial clustering based on discrete genetic structure (Fig. 2B), that is, green, blue, and red locations appeared intermixed (Fig. 2B). Furthermore, ellipses to delineate the spatial extent of possible subpopulations based on genetic clusters were overlapping (Fig. 2B). There was little differentiation between pairwise mean *F*_ST_ (group 1 versus group 2: 0.021; group 1 versus group 3: 0.016; and group 2 versus group 3: 0.022). Subsequent amova analysis revealed that a small (2.7%) but significant (*P* < 0.0001) amount of genetic variation was related to differences among clusters.

**Figure 3:**
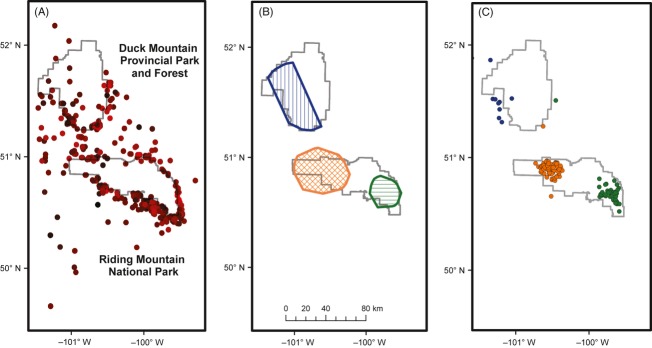
Spatial information on genotyped white-tailed deer (A) analysis of global eigenvalue scores for the spatial principal component analyses (sPCA) performed on the 494 genotyped white-tailed deer (2004–2006). All plots are positioned according to their spatial coordinates. Shades of red represent genetic distance where the more related two individuals were the more similar their color; for example, an individual depicted as dark red is closer genetically to an individual that is a similar shade of red than an individual whom is depicted as light red. No clustering in shading revealed no discernible spatial patterning on the landscape. We contrast the absence of spatial structure in white-tailed deer against three elk clusters that have been extrapolated from radio-telemetry relocation data (*n =* 11 194 over *n* = 379 collared elk collected between 2002–2009) to illustrate the distribution of bounded subpopulations of elk ([B] calculated using 95% minimum convex polygons). Furthermore, spatial structure from radio-telemetry data correlates with spatial information on genotyped elk ([C] *n =* 312 at 30 microsatellites) from Riding Mountain National Park which occupy three spatially distinct clusters (Vander Wal et al. 2012).

Spatial Principal Component Analysis (sPCA) scores can detect clines and spatial groups (global structures) as well as strong genetic differences between neighbors (local structures). A global permutation test on the eigenvalues derived from a sPCA with a minimum neighbor connection network did not find any significant global or local structures (*P*_GLOBAL_ = 0.1, *P*_LOCAL_ = 0.6). The first two eigenvalues were large compared with the others, and therefore were retained. Individual scores on these two axes are shown on Fig. 3A. Had spatial structure been detected with the sPCA similarly shaded white-tailed deer locations would have clustered together or alternatively would appear clinal across the landscape from light to dark red. However, individual scores did not show any sharp boundaries or any progressive change. Ultimately, sPCA, DAPC [and STRUCTURE (Pritchard et al. 2000), *K* = 1 data are not shown] all corroborated the lack of any spatial structure in the white-tailed deer dataset.

### Elk: telemetry and spatial structure

Radio-telemetry relocations segregated the regional population of elk into three nonoverlapping geographic areas (95% minimum convex polygons, Fig. 3B), where elk were most commonly relocated. Thus, 95% of all animal relocations have occurred within each of these areas. Because we never documented collared animals moving among areas, we inferred that such movements were highly unlikely. The geographic locations of these polygons highlighted that these distinct areas corresponded to that of the cryptic population structure revealed by our previous landscape genetic analyses [(Vander Wal et al. 2012) and Fig. 3C]. At the landscape-scale, radio-telemetry and sPCA analyses revealed that the elk population is composed of three spatial clusters. These clusters corresponded to discrete areas within a southeast to northwest genetic cline of elk (Vander Wal et al. 2012). Within this cline, which spans across the two protected areas present in the region, occurs a genetically distinct cluster corresponding to the west RMNP subpopulation. (Fig. 3B,C, see also Vander Wal et al. 2012).

### Dispersion of bovine tuberculosis-positive white-tailed deer and elk

**Figure 4:**
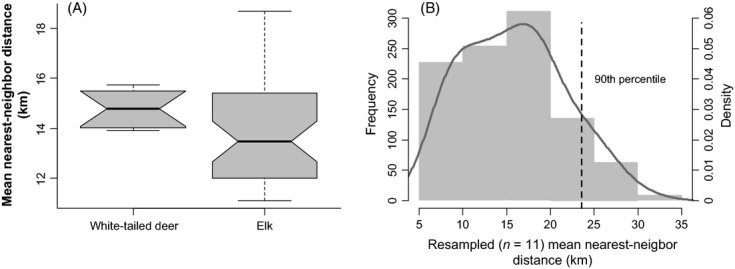
Comparison of nearest-neighbor distances between bovine tuberculosis (bTB)-positive white-tailed deer and elk, illustrating some support that bTB-positive white-tailed deer may be more dispersed. Boxplots (A) (median, 25% and 75% quartiles, and 95% confidence intervals) illustrating that despite small sample size, white-tailed deer with bovine tuberculosis (*n* = 11) were more dispersed on the landscape than bovine tuberculosis-positive elk (*n =* 41). Nonoverlapping notches (‘> <‘) indicate that the two groups are likely significantly different (Chambers et al. 1983). This is confirmed by a Mann–Whitney *U*-test (χ = 4.94, *P =* 0.02, df = 1). A histogram (B) with smoothed density distribution (solid curve) illustrating that mean nearest-neighbor distances for white-tailed deer fall at the 90th percentile (dashed line) of 1000 iterations of an *n* = 11 bootstrap (without replacement) of bTB-positive elk mean nearest-neighbor distances.

The Riding Mountain region has low bTB prevalence and only 11 white-tailed deer and 41 elk were bTB positive (i.e., infected). Infected white-tailed deer appeared to be more dispersed than infected elk. For the Mann–Whitney *U*-test, bTB-positive white-tailed deer were significantly more dispersed than bTB-positive elk (Fig. 4A, χ = 4.94, *P* = 0.02, df = 1). However, the bootstrap results were not significant at *P =* 0.05. Rather the average mean nearest-neighbor distance for white-tailed deer occurred at the 90th percentile of the distribution of resampled elk mean nearest-neighbor distances (Fig. 4B).

## Discussion

Differences in connectivity among subpopulations are known to affect the probability of landscape-scale disease transmission (Hess 1994; Collinge et al. 2005; Real and Biek 2007). Here, we demonstrate that at the landscape-scale sympatric populations of white-tailed deer and elk show contrasting populations structures. Although the elk population is spatially structured with low connectivity among subpopulations, the white-tailed deer is relatively uniform. This difference mirrors our finding that infected (bTB positive) white-tailed deer individuals are more dispersed on the landscape than infected elk. Overall our results indicate that white-tailed deer is the host more likely to be involved in long-distance transmission of the disease.

In the Riding Mountain Region, elk have the highest prevalence of bTB and have traditionally been considered the reservoir and main potential vector for the spread of *M. bovis* into neighboring cattle farms (Brook and McLachlan 2006, 2009). However, fragmentation and loss of wildlife corridors (Walker 2001) have reduced the capability of elk to disperse diseases across the landscape into other subpopulations. Moreover, our genetic and biotelemetry data clearly show that the remnant elk population in the RMNP region consists of three spatially discrete subpopulations (Fig. 3B,C), one of which is currently endemic with bTB (Fig. 1, Vander Wal et al. 2012). Thus, direct transmission of bTB among subpopulations of elk or between elk and cattle farms not proximal to the bTB infection focus is unlikely due to the observed bounded spatial structure and uncommon elk dispersals (Vander Wal et al. 2012).

In comparison with elk, white-tailed deer are well adapted to highly modified agricultural landscapes (Côté et al. 2004). This has allowed white-tailed deer to extend their geographic range west and north following European colonization of the Prairie and Boreal Plain ecoregions (Wishart 1984; Côté et al. 2004; McShea 2012). Consequently, in our study region, white-tailed deer are more widely distributed and more abundant than elk (Brook 2008). This coupled with the fact that white-tailed deer are much more likely to interact with cattle than elk (Brook et al. 2012) highlights the risk posed by white-tailed deer for the spread of bTB on the landscape and to cattle. Our results suggest that this may be the case. Our population genetic analyses revealed the lack of physiognomic landscape features that act as insurmountable barriers to white-tailed deer dispersal (Ellsworth et al. 1994; Rogers et al. 2011; Lang and Blanchong 2012; Robinson et al. 2012). However, the population is not necessarily panmictic. Although not spatially structured, white-tailed deer fall into three genetically distinct sympatric clusters (Fig. 2). Pairwise mean *F*_ST_ suggests mixing but there is still a small (2.7%) amount of genetic variation retained among clusters. We did not, however, sequence mtDNA haplotypes and cannot comment on whether these clusters represent vestiges of the colonization or expansion of distinct geographic lineages. Nor do we have information to suggest that assortative mating contributes to the maintenance of sympatric clusters. Notwithstanding this, our results underscore the high connectivity that characterizes white-tailed deer populations.

Because of being highly connected, white-tailed deer may disperse bTB further from the bTB infection focus than elk. Our dispersion results are not fully conclusive as the mean distance to the nearest infected neighbor for white-tailed deer occurred at the 90th percentile of the bootstrapped mean infected-neighbor distance distribution for elk. Despite the intensive sampling (6,909 white-tailed deer and 3,620 elk, Shury and Bergeson 2011), the low bTB prevalence translates into low sample sizes for infected animals (*n*_white-tailed deer_ = 11, *n*_elk_ = 41). Furthermore, appreciating that these samples were collected using mixed sampling methods (see *General Sampling Considerations*). Thus, our results should be viewed as a trend, rather than a definitive result.

For white-tailed deer to act as vectors from the focal area of bTB among uninfected subpopulations of elk (and among cattle herds), transmission needs to occur interspecifically. This can occur from animal to animal and by environmental contamination (Williams et al. 2002; Palmer et al. 2004; Mathiason et al. 2006). Although much remains unknown about interspecific transmission between cervids (but see Hamir et al. 2011), two indirect lines of evidence suggest that it occurs locally. First, transmission via the environment is likely due to white-tailed deer and elk sharing similar space and food sources (Conover 1997; Beck and Peek 2005; Jenkins et al. 2011; Walter et al. 2011). Rudolph et al. (2006) found that bTB transmission risk increases at hunter bait sites, even if the survival of *M. bovis* in the environment is relatively short (Duffield and Young 1985). Second, the same endemic spoligotype of bTB has been detected in white-tailed deer, elk, and cattle in the Riding Mountain Region (Lutze-Wallace et al. 2005). Interspecific transmission may have contributed to the evolution of bTB strains in Manitoba, which are considered endemic (Lutze-Wallace et al. 2005), this despite the origins of bTB being non-North American in origin (Mostowy and Behr 2005) and that bTB in the RMNP cervids was likely historically and perhaps contemporarily acquired from cattle that frequently grazed within the current boundaries of the park (Brook 2009).

We have highlighted that understanding the potential for pathogen transmission among wildlife at landscape scales requires knowledge of the population structures of all free-ranging hosts, particularly when connectivity among populations can be a function of intraspecific and/or heterospecific dispersals. Our study suggests that the role of white-tailed deer in bTB transmission is likely to be more critical than previously appreciated. This has important applications for ongoing intervention programs that so far have been largely elk-biased. Several behavioral and life history traits make managing disease in white-tailed deer especially problematical. For example, white-tailed deer are more likely to co-mingle with cattle on winter cattle feeding areas (Brook et al. 2012). White-tailed deer also aggregate in ‘winter yards’, a network of packed trails to minimize effort traveling through deep snow. Winter yards may play an important role in amplifying intraspecific transmission (Lankester and Peterson 1996) and landscape-level transmission because they concentrate individuals, which subsequently disperse after snow melt. Either sharing these areas with elk or moving from winter yards to areas shared by elk have the potential to exacerbate interspecific transmission. Life history traits that are problems for managing disease in white-tailed deer include their often extremely high population density, high reproductive rates, and high dispersal rates (Côté et al. 2004). These problems, often seen in single host-pathogen systems, for example, white-tailed deer and bTB in Michigan (O'Brien et al. 2004) and white-tailed deer and CWD in Wisconsin (Joly et al. 2006), are further compounded when multiple hosts are present, as is the case for bTB in the Riding Mountain Region.

## Data archiving statement

Data for this study are available as supplementary material online.

## Supporting Information

**Table S1.** It includes data on Locus No, Locus, Source, Lane, Proportion of Missing Data, Min, Max, Primers, No. of Alleles, He and Ho and HWE.

**Table S2.** Microsatellite data used for white-tailed deer landscape genetics.
