# Supplementary material for: Juxtaposition between host population structures: implications for disease transmission in a sympatric cervid community
Source: Evol Appl. 2013 Oct 9;6(7):1001–11. doi: 10.1111/eva.12065 (PMC3804234; doi:10.1111/eva.12065)
Supplement: Supplementary file 1 [file eva0006-1001-SD1.docx]

**Supplementary Material:** *Juxtaposition between host population structures: implications for disease transmission in a cervid community*, Vander Wal et al. Supplementary Table 1. Microsatellite data used for white-tailed deer landscape genetics.

| **Locus No.** | **Locus** | **Source** | **Lane^1^** | **Proportion of missing data** | **Min** | **Max** | **Primers** | **No. Alleles** | ***He*^2^** | ***HWE*^3^** |
| --- | --- | --- | --- | --- | --- | --- | --- | --- | --- | --- |
|  |  |  |  |  |  |  |  |  | ***Ho*** |  |
| L01 | **BBJ2** | (Wilson & Strobeck 1999) | 1 | 0.0061 | 126 | 146 | ACA CTG CCC CGG TAT CTT TG | 9 | 0.50 | *P =* 1 |
|  |  |  |  |  |  |  | GCA CTT TAG CTC ACT TCC TG |  | 0.52 |  |
| L02 | **BM415** | (Bishop et al. 1994) | 1 | 0.2611 | 139 | 149 | GCT ACA GCC CTT CTG GTT TG | 5 | 0.37 | *P* = 0.0005 |
|  |  |  |  |  |  |  | GAG CTA ATC ACC AAC AGC AAG |  | 0.72 |  |
| L03 | **Cervid1** | (DeWoody, Honeycutt, & Skow 1995) | 1 | 0.0142 | 159 | 193 | AAATGACAACCCGCTCCAGTATC | 16 | 0.79 | *P* = 1 |
|  |  |  |  |  |  |  | GTTTCCGTGCATCTCAACATGAGTTAG |  | 0.812 |  |
| L04 | **INRA011** | (Vaiman et al. 1992) | 1 | 0.004 | 191 | 205 | CGAGTTTCTTTCCTCGTGGTAGGC | 9 | 0.471 | *P* = 0.9725 |
|  |  |  |  |  |  |  | GCTCGGCACATCTTCCTTAGCAAC |  | 0.49 |  |
| L05 | **OCAM** | (Moore et al. 1992) | 1 | 0.0466 | 193 | 211 | CCTGACTATAATGTACAGATCCTC | 12 | 0.81 | *P* = 1 |
|  |  |  |  |  |  |  | GCAGAATGACTAGGAAGGATGGCA |  | 0.83 |  |
| L06 | **R** | (Jones, Levine, & Banks 2000) | 1 | 0.0142 | 249 | 259 | GGGGTCTTCTCAATCCA | 5 | 0.55 | *P* = 0.744 |
|  |  |  |  |  |  |  | TCAGTTGGAACTCTAAAGT |  | 0.55 |  |
| L07 | **Rt30** | (Wilson et al. 1997) | 1 | 0.0587 | 103 | 133 | GTG TAA CCC AAA GGG CAA CT | 19 | 0.38 | *P* = 0.0135 |
|  |  |  |  |  |  |  | CTG GTG TAT GTA TGC ACA CT |  | 0.88 |  |
| L08 | **BL25** | (Bishop et al. 1994) | 2 | 0.0142 | 174 | 180 | AACAGTGGCAATGGAAGTGG | 6 | 0.46 | *P* = 0.1085 |
|  |  |  |  |  |  |  | AGTCAGGATCTAGTGGGTGAGTG |  | 0.49 |  |
| L09 | **BM4208** | (Bishop et al. 1994) | 2 | 0.0243 | 80 | 116 | TCA GTA CAC TGG CCA CCA TG | 16 | 0.77 | *P* = 0.4300 |
|  |  |  |  |  |  |  | CAC TGC ATG CTT TTC CAA AC |  | 0.85 |  |
| L10 | **BM6438** | (Bishop et al. 1994) | 2 | 0.0506 | 248 | 274 | TTGAGCACAGACACAGACTGG | 15 | 0.85 | *P* = 1 |
|  |  |  |  |  |  |  | ACTGAATGCCTCCTTTGTGC |  | 0.88 |  |
| L11 | **BM848** | (Bishop et al. 1994) | 2 | 0.1478 | 361 | 383 | TGGTTGGAAGGAAAACTTGG | 14 | 0.70 | *P* = 1 |
|  |  |  |  |  |  |  | CCTCTGCTCCTCAAGACAC |  | 0.77 |  |
| L12 | **BovPRL** | (Moore et al. 1992) | 2 | 0.0061 | 157 | 159 | GGA AAG TGA ACA TGA CTG TCT AG | 2 | 0.40 | *P* = 0.3445 |
|  |  |  |  |  |  |  | GCC CTC TCT TCT ACA ATG AAC AC |  | 0.42 |  |
| L13 | **K** | (Jones et al. 2000) | 2 | 0.004 | 113 | 129 | GCAGGAAGGAGGAGACAGTA | 7 | 0.40 | *P* = 1 |
|  |  |  |  |  |  |  | GCTGGTTCGTTATCATTTAGC |  | 0.38 |  |
| L14 | **O** | (Jones et al. 2000) | 2 | 0.1518 | 274 | 292 | ACGAGGTTCAGTGGTTCC | 6 | 0.41 | *P* = 0.2695 |
|  |  |  |  |  |  |  | CAGGGCATAGTTTCCAAA |  | 0.54 |  |
| L15 | **Rt7** | (Wilson et al. 1997) | 2 | 0.0202 | 208 | 240 | CCT GTT CTA CTC TTC TTC TC | 16 | 0.83 | *P* = 1 |
|  |  |  |  |  |  |  | ACT TTT CAC GGG CAC TGG TT |  | 0.86 |  |
| L16 | **BM1225** | (Bishop et al. 1994) | 3 | 0.087 | 227 | 253 | ACC CCT ATC ACC ATG CTC TG | 9 | 0.58 | *P* = 0.003 |
|  |  |  |  |  |  |  | TTT CTC AAC AGA GGT GTC CAC |  | 0.74 |  |
| L17 | **BM4107** | (Bishop et al. 1994) | 3 | 0.0506 | 157 | 191 | AGC CCC TGC TAT TGT GTG AG | 17 | 0.74 | *P* = 1 |
|  |  |  |  |  |  |  | ATA GGC TTT GCA TTG TTC AGG |  | 0.82 |  |
| L18 | **BM6506** | (Bishop et al. 1994) | 3 | 0.0263 | 264 | 280 | GCACGTGGTAAAGAGATGGC | 13 | 0.72 | *P* = 0.006 |
|  |  |  |  |  |  |  | AGCAACTTGAGCATGGCAC |  | 0.87 |  |
| L19 | **D** | (Jones et al. 2000) | 3 | 0.0385 | 150 | 190 | AGAGCCTCGTCTTTTCATTC | 12 | 0.54 | *P* = 0.0615 |
|  |  |  |  |  |  |  | TTGCTGCTTGCTTGTCTAAT |  | 0.81 |  |
| L20 | **Eth152** | (Steffen et al. 1993) | 3 | 0.0972 | 172 | 198 | AGGGAGGGTCACCTCTGC | 15 | 0.67 | *P* = 1 |
|  |  |  |  |  |  |  | CTTGTACTCGTAGGGCAGGC |  | 0.79 |  |
| L21 | **N** | (Jones et al. 2000) | 3 | 0.0162 | 288 | 336 | TCCAGAGAAGCAACCAATAG | 23 | 0.81 | *P* = 1 |
|  |  |  |  |  |  |  | GTGTGCCTTAAACAACCTGT |  | 0.89 |  |
| L22 | **OarFCB193** | (Buchanan & Crawford 1993) | 3 | 0.0243 | 96 | 124 | TTCATCTCAGACTGGGATTCAGAAAGGC | 15 | 0.86 | *P* = 1 |
|  |  |  |  |  |  |  | GCTTGGAAATAACCCTCCTGCATCCC |  | 0.84 |  |
| L23 | **P** | (Jones et al. 2000) | 3 | 0.0466 | 208 | 240 | TTTCACTGTTTTCTCCTTCAGA | 14 | 0.52 | *P* = 0.0005 |
|  |  |  |  |  |  |  | GTTTCTTTGCCCAATCAGATGTTGTAG |  | 0.83 |  |
| L24 | **Rt5** | (Wilson et al. 1997) | 3 | 0.0263 | 84 | 97 | CAG CAT AAT TCT GAC AAG TG | 17 | 0.82 | *P* = 1 |
|  |  |  |  |  |  |  | GTT GAG GGG ACT CGA CTG |  | 0.84 |  |

^1^ Loci that were run together in the same lane.

^2^ Expected heterozygosity (*He*) / Observed heterozygosity (*Ho*)

**^3^** Whether the loci differs significantly from Hardy-Weinberg equilibrium according to a permuted *Chi* square test (Jombart 2008).

References:

Bishop, M.D., Kappes, S.M., Keele, J.W., Stone, R.T., Sunden, S., Hawkins, G.A., Toldo, S.S., Fries, R., Grosz, M.D., Yoo, J. & Beattie, C.W. (1994) A genetic linkage map for cattle. *Genetics*, **136**, 619 –639.

Buchanan, F.C. & Crawford, A.M. (1993) Ovine mirosatellites at the OarFCB11, OarFCB128, OarFCB193, OarFCB266, and OarFCB204 loci. *Animal Genetics*, **24**, 145.

DeWoody, J.A., Honeycutt, R.L. & Skow, L.C. (1995) Microsatellite markers in white-tailed deer. *Journal of Heredity*, **86**, 317 –319.

Jombart, T. (2008) adegenet: a R package for the multivariate analysis of genetic markers. *Bioinformatics*, **24**, 1403–1405.

Jones, K.C., Levine, K.F. & Banks, J.. (2000) DNA- based genetic markers in black- tailed and mule deer for forensic applications. *California Fish and Game*, **86**, 115–126.

Moore, S.S., Barendse, W., Berger, K.T., Armitage, S.M. & Hetzel, D.J.S. (1992) Bovine and ovine DNA microsatellites from the EMBL and GENBANK databases. *Animal Genetics*, **23**, 463–467.

Steffen, P., Eggen, A., Stranzinger, G., Fries, R., Dietz, A.B. & Womack, J.E. (1993) Isolation and mapping of polymorphic microsatellites in cattle. *Animal Genetics*, **24**, 121–124.

Vaiman, D., Osta, R., Mercier, D., Grohs, C. & Leveziel, H. (1992) Characterization of five new bovine dinucleotide repeats. *Animal Genetics*, **23**, 537–541.

Wilson, G.A. & Strobeck, C. (1999) Genetic variation within and relatedness among wood and plains bison populations. *Genome*, **42**, 483–496.

Wilson, G.A., Strobeck, C., Wu, L. & Coffin, J.W. (1997) Characterization of microsatellite loci in caribou Rangifer tarandus, and their use in other artiodactyls. *Molecular Ecology*, **6**, 697–699.
